# Time-Course Transcriptome Analysis of Lungs From Mice Infected With Hypervirulent *Klebsiella pneumoniae via* Aerosolized Intratracheal Inoculation

**DOI:** 10.3389/fcimb.2022.833080

**Published:** 2022-04-27

**Authors:** Xinying Zheng, Jianshu Guo, Chaoyue Cao, Tongtong Qin, Yue Zhao, Xiaolin Song, Meng Lv, Lingfei Hu, Lili Zhang, Dongsheng Zhou, Tongyu Fang, Wenhui Yang

**Affiliations:** ^1^College of Life Science and Technology, Beijing University of Chemical Technology, Beijing, China; ^2^State Key Laboratory of Pathogen and Biosecurity, Beijing Institute of Microbiology and Epidemiology, Beijing, China; ^3^Laboratory Animal Center, Academy of Military Medical Sciences, Beijing, China

**Keywords:** hypervirulent *Klebsiella pneumoniae*, molecular pathology, lung, time-course transcriptome, inflammation

## Abstract

Hypervirulent *Klebsiella pneumoniae* (hvKp) can cause life-threatening community-acquired infections among healthy young individuals and is thus of concern for global dissemination. In this study, a mouse model of acute primary hvKp pneumonia was established *via* aerosolized intratracheal (i.t.) inoculation, laying the foundation for conducting extensive studies related to hvKp. Subsequently, a time-course transcriptional profile was created of the lungs from the mouse model at 0, 12, 24, 48 and 60 hours post-infection (hpi) using RNA Sequencing (RNA-Seq). RNA-Seq data were analyzed with the use of Mfuzz time clustering, weighted gene co-expression network analysis (WGCNA) and Immune Cell Abundance Identifier for mouse (ImmuCellAI-mouse). A gradual change in the transcriptional profile of the lungs was observed that reflected expected disease progression. At 12 hpi, genes related to acute phase inflammatory response increased in expression and lipid metabolism appeared to have a pro-inflammatory effect. At 24 hpi, exacerbation of inflammation was observed and active IFN-γ suggested that signaling promoted activation and recruitment of macrophages occurred. Genes related to maintaining the structural integrity of lung tissues showed a sustained decrease in expression after infection and the decrease was especially marked at 48 hpi. TNF, IL-17, MAPK and NF-kB signaling pathways may play key roles in the immunopathogenesis mechanism at all stages of infection. Natural killer (NK) cells consistently decreased in abundance after infection, which has rarely been reported in hvKp infection and could provide a new target for treatment. Genes *Saa1* and *Slpi* were significantly upregulated during infection. Both *Saa1*, which is associated with lipopolysaccharide (LPS) that elicits host inflammatory response, and *Slpi*, which encodes an antimicrobial protein, have not previously been reported in hvKp infections and could be important targets for subsequent studies. To t our knowledge, this paper represents the first study to investigate the pulmonary transcriptional response to hvKp infection. The results provide new insights into the molecular mechanisms underlying the pathogenesis of hvKp pulmonary infection that can contribute to the development of therapies to reduce hvKp pneumonia.

## Introduction

*Klebsiella pneumoniae* is a Gram-negative commensal bacterium and opportunistic pathogen found ubiquitously in the environment. It often colonizes on human mucosal surfaces and causes various nosocomial infections in the respiratory tract, lung, urinary tract, wound sites and blood (Podschun and Ullmann, 1998). Moreover, it is one of the few Gram-negative bacteria capable of causing primary pneumonia (Restuccia and Cunha, 1984) and is a major cause of hospital-acquired pneumonia (Magill et al., 2018). *K. pneumoniae* strains are classified into two distinct pathotypes: classic *K. pneumoniae* (cKp) and hypervirulent *K. pneumoniae* (hvKp) (Catalán-Nájera et al., 2017; Russo and Marr, 2019). The majority of infections caused by *K. pneumoniae* come from the “classical” strains of cKp (Shon et al., 2013). In contrast to cKp, hvKp is more virulent and capable of causing severe organ or life-threatening infections, such as pneumonia, hepatic abscess, meningitis and necrotizing fasciitis, in healthy individuals from community settings (Fang et al., 2007; Lederman and Crum, 2005; Patel et al., 2014; Pomakova et al., 2012). Although community-acquired pneumonia (CAP) due to hvKp is uncommon (Choby et al., 2020), it is noteworthy that bacterial CAP due to hvKp has higher mortality and respiratory failure rates compared with *Streptococcus pneumoniae*, the leading cause of CAP globally (Lin et al., 2010). In the past few decades, hvKp has spread globally and the incidence of infections has been increasing steadily (Lederman and Crum, 2005; Pomakova et al., 2012). Hence, a better understanding of hvKp pulmonary infection is warranted.

Mechanisms of hvKp infection pathogenesis and hvKp-host interactions are complex. Lipopolysaccharide (LPS), a major component of the outer membrane of *K. pneumoniae* (Whitfield and Trent, 2014), is recognized by the key pattern recognition receptor, toll-like receptor 4 (TLR4), which triggers the innate immune response (Akira et al., 2006; Scott et al., 2017). One of the most prominent bacterial phenotypes associated with hvKp is overproduction of capsule polysaccharide (CPS) (Yu et al., 2008), which leads to a hypermucous phenotype. Overproduction of CPS impairs complement-mediated bacterial killing and phagocytosis *via* neutrophils and macrophages, which directly correlates with host resistance to hvKp (Cheng et al., 2010; Catalina et al., 2013). Macrophages are essential for controlling *K. pneumoniae* replication and regulating the inflammatory response at different tissue sites, such as the lung, liver and spleen (Olonisakin et al., 2021). Monocytes are heterogeneous cells capable of displaying proinflammatory or immuno-regulatory phenotypes, depending on the nature of the microenvironment at the site of infection (Peñaloza et al., 2019). Inflammatory monocytes are required to clear *K. pneumoniae* from the lung (Xiong et al., 2016), while anti-inflammatory monocytes are recruited to the lung at later stages of *K. pneumoniae* infection and appear to play a beneficial role by mediating the clearance of apoptotic neutrophils (Poe et al., 2013). Overall, these results indicate that hvKp infection may induce discriminatory gene expression patterns in different types of host cells with different effects on specific cell types.

The lung is both a major immune organ in vertebrates and an important target organ for hvKp infection. Thus, a detailed description of hvKp pulmonary infection is necessary to explore the mechanisms of hvKp-host interactions. RNA-Seq, a recently developed method for high-throughput transcriptome sequencing (Hong et al., 2017), can reveal dynamic changes in host gene expression during pathogen infection and has been used to study various viral infections and diseases (He et al., 2017; Arun et al., 2018). Based on the above, we performed time-course RNA sequencing of lung tissues from mice with primary hvKp pneumonia to investigate transcription profile changes in hvKp-infected lungs and to screen for genes or other valuable research targets. This study promotes our understanding of pathogenesis associated with hvKp pulmonary infection.

## Materials and Methods

### Bacterial Strain and Growth Conditions

While no molecular diagnostic nor microbiological consensus exists for the definition of hypervirulence (Harada and Doi, 2018; Shan et al., 2006), the NTUH-K2044 hvKp strain has been recognized as hypervirulent given it possesses the *magA* and *rmpA* genes, belongs to capsular serotype K1, has high virulence and hypermucoviscosity (Fang et al., 2004; Chuang et al., 2006; Yu et al., 2006; Yeh et al., 2007). In this study, hvKp strain NTUH-K2044 was inoculated into Brain Heart Infusion (BHI) broth (BD Biosciences, Lawrence, KS) and grown overnight at 37°C with continuous shaking at 220 rpm. The overnight culture was diluted with BHI at 1:200 and incubated at 37°C with shaking for 3 h to achieve OD_600_ = 1.5. Then the culture was further diluted and incubated for another 2.5 h. The final culture was centrifuged, washed and resuspended in phosphate-buffered saline (PBS) to achieve OD_600_ = 1.0, a concentration of about 6 x 10^8^ CFU/mL. The actual infection dose in each experiment was determined by serial dilution plating on BHI agar.

### Mice Infection

Female C57BL/6Cnc mice aged 6-8 weeks were purchased from Vital River Laboratories (Beijing, China). Mice were exposed to aerosolized hvKp (2 x 10^4^ CFU in 50 μL PBS per mouse) *via* aerosolized intratracheal inoculation as previously described (Feng et al., 2019). Briefly, each mouse was deeply anesthetized with pentobarbital sodium, a Micro Sprayer (Huironghe Company, Beijing, China) was inserted into the tracheal bifurcation of the mouse and hvKp aerosol was then generated by the Micro Sprayer and sprayed into the lung. Following exposure, survival was monitored twice daily for 14 days. Animals were randomly divided into four infection groups and one control group (five mice per group), and the infected mice were euthanized at 12, 24, 48 and 60 h post-infection (hpi) while the control group mice were euthanized immediately after delivery of PBS at the 0 h time point. The lungs of the mice were isolated and divided into two parts for subsequent histopathologic examination or total RNA extraction. All animal experiments were performed in the Laboratory Animal Center of Academy of Military Medical Science (AMMS), approved by the Animal Care and Use Committee (IACUC) of AMMS, and the ethical approval number was IACUC-DWZX-2020-050.

### Histopathological Validation

Collected lungs were fixed in 4% paraformaldehyde, and the fixed tissues sliced, mounted on slides, and stained with hematoxylin-eosin (HE). Pathological changes in tissue slices were observed by light microscopy (BX60, Olympus, Japan). Tissue sections were evaluated by a trained pathologist (blind to treatment) according to the following scores: 0, no pathological lesions; 1, minimal; 2, mild; 3, moderate; 4, severe. The degree of pathological lesions was related to the distribution of lesions as follows: inflammatory cell infiltration, edema, congestion, and tissue necrosis. Pathology scores were assessed using one-way multilevel ANOVA in SAS 9.3.

### RNA Extraction, Library Preparation, and Sequencing

The collected lungs were submerged in RNA*later* stabilization solution (Invitrogen, Carlsbad, CA, USA) and total RNA was extracted using an RNA purification kit (Invitrogen, Carlsbad, CA). The concentration and purity of the extracted RNA were measured using a Nanodrop 2000c spectrophotometer (Thermo Fisher, Waltham, Massachusetts, USA). Library construction and sequencing were conducted by the Novogene Company in Beijing, China. No less than 1 μg of RNA per sample was used as the input material for RNA sample preparation. Sequencing libraries were generated using NEBNext^®^ Ultra TMRNA Library Prep Kit for Illumina^®^ (NEB, Ipswich, MA, USA). The library preparations were sequenced using the Illumina sequencing platform (HiSeq™ 2500, Illumina, San Diego, California, USA), sequenced reads from all samples were mapped to the reference genome (mouse) using Hisat2 v2.0.5, and then fragments per kilobase of exon per million fragments mapped (FPKM) values were calculated for each gene based on gene length. FPKM (Florea et al., 2013) was used as the unit of measurement to estimate transcript abundance.

### Identification of DEGs and Bioinformatics Analysis

To normalize the RNA-Seq data, differential expression analysis was performed using the edgeR package in R (Smyth, 2010). Triplicates of the RNA-Seq experiments were analyzed separately and resulting *p*-value were adjusted using Benjamini and Hochberg’s approach for controlling false discovery rate (FDR) (Love et al., 2014). The selection criteria for differentially expressed genes (DEGs) in this study was an adjusted *p*–value <0.05 and an absolute fold change >2 or <0.5. To assess quality of the data, principal component analysis (PCA) was used to examine the distribution of samples. Based on the Gene Ontology (GO) database (http://geneontology.org/) and Kyoto Encyclopedia of Genes and Genomes (KEGG) database (https://www.genome.jp/kegg/), the Clusterprofiler (Guangchuang et al., 2012) package of R was applied to perform functional enrichment and pathway analysis, with corrected *p*-value <0.05 considered significantly enriched.

### Time Series Gene Clustering

Soft cluster analysis using the fuzzy C-Means algorithm in the Mfuzz package (Futschik and Carlisle, 2011; Kumar and Futschik, 2007) was conducted to assign genes to clusters according to the expression pattern of DEGs. The number of clusters was set to nine and the fuzzifier coefficient, M, was set to 1.71.

### Weighted Gene Co-Expression Network Analysis (WGCNA)

To identify modules with different expression patterns, a weighted correlation network analysis (WGCNA) was conducted for the 15 lung samples using the WGCNA package (Langfelder and Horvath, 2009) in R. The WGCNA input data were normalized values for each transcript and correlations between any two genes were first collected and analyzed by Pearson correlation coefficients to form a similarity matrix (Ivliev et al., 2010). At the same time, the topological overlap matrix (TOM) method was employed to take both direct and indirect relationships into account. Then, the hierarchical clustering tree was used to generate a division of gene modules based on the TOM values between genes. The module with the highest correlation for the sample characteristics was selected for further analysis.

### Immune Infiltration Analysis

Immune Cell Abundance Identifier (ImmuCellAI) is a tool to accurately estimate immune cell abundance from gene expression datasets, including RNA-Seq and microarray data (Miao et al., 2020). Immune Cell Abundance Identifier for mouse (ImmuCellAI-mouse) is used as a complement to ImmuCellAI to estimate the abundance of 36 immune cell (sub)types in mouse transcriptome data (Miao et al., 2021). ImmuCellAI-mouse simulated the process of flow cytometry analysis by adopting a hierarchical strategy, dividing 36 cell types into three layers. Layer 1 is composed of seven major immune cell types: B cells, monocytes, dendritic cells (DCs), natural killer (NK) cells, granulocytes, macrophages and T cells. Cells in layer 2 are subtypes of cells in the first layer, including subtypes of B cells (B1, follicular B, germinal center B, marginal zone B, memory B and plasma B cells), subtypes of DCs (cDC1, cDC2, MoDC and pDC cells), subtypes of granulocytes (basophil, eosinophil, mast cell and neutrophils), subtypes of macrophages (M1 and M2 macrophages) and subtypes of T cells (CD4 T, CD8 T, NKT and gamma-delta T cells). Finally, cells in layer 3 are subtypes of CD4 T and CD8 T cells (including CD4+ naïve, CD4+ memory, Treg, T helper, CD8+ naïve, CD8+ central memory, CD8+ effector memory, cytotoxic and exhausted cells).

### Validation of RNA-Seq by qRT-PCR

To validate the results of the RNA-Seq data, 12 DEGs were randomly selected for qRT-PCR validation based on their expression patterns at four time points. RNA samples were reverse-transcribed into cDNA using the TransScript One-Step gDNA Removal and cDNA Synthesis SuperMix (TransGen Biotech, Beijing, China). Primers used in this study have been previously published or can be found in PrimerBank (https://pga.mgh.harvard.edu/primerbank/); primer sequences are listed in **Table 1**. qRT-PCR was performed using the LightCycler 96 RT-PCR Detection System (Roche, Basel, Switzerland). Each reaction mixture was 20 μL in total, containing 10 μL of SYBR qPCR Master Mix (QIAGEN, Dusseldorf, Germany), 0.7 μL of each upstream and downstream primers (7 μM), 1 μL of cDNA template, and 7.6 μL of ddH_2_O. The following reaction procedure was used: 95°C for 120 s, then 42 cycles at 95°C for 15 s and 60°C for 30 s. Each experiment was performed in triplicate. The relative expression values of selected genes were calculated using the 2^-ΔΔCt^ method and normalized against expression levels of the β-actin gene. Correlation between the RNA-Seq and qRT-PCR data was analyzed using Pearson’s correlation coefficient.

**Table 1:** Primers sequences used for qRT-PCR in this study.

Gene	Forward primer sequence	Reverse primer sequence
AA467197	5’-ATCTTTCGCTTTGTATGCGTTGA- 3’	5’-GGCTTCCATTGCTGGTTGATG- 3
Adamts4	5’-ATGGCCTCAATCCATCCCAG- 3	5’-AAGCAGGGTTGGAATCTTTGC- 3’
Cyp27a1	5’-CCAGGCACAGGAGAGTACG- 3’	5’-GGGCAAGTGCAGCACATAG- 3’
Faim2	5’-GACCCCAGACATCACGAGC- 3’	5’-GGTTAGCCTGGACATAGTCCTTA- 3’
Igsf6	5’-TTCCAAGTCGGTATGGTGGGT- 3’	5’-CGAAACCACAAGCTCTTTGGTG- 3’
Serpine1	5’-TTCAGCCCTTGCTTGCCTC- 3’	5’-ACACTTTTACTCCGAAGTCGGT- 3’
Fgfr4	5’-TTGGCCCTGTTGAGCATCTTT- 3’	5’-GCCCTCTTTGTACCAGTGACG- 3’
Hpcal4	5’-CTTCGAGCAGAAGCTCAACTG- 3’	5’-TGCCCACCATCTTATAGATAGCC- 3’
Timp1	5’-GCAACTCGGACCTGGTCATAA- 3’	5’-CGGCCCGTGATGAGAAACT- 3’
Colq	5’-TCCTGGCCTGGATCAGAAGAA- 3’	5’-GGTGATGGTGACGCCTCAA- 3’
Lbh	5’-CTGCTCTGACTATCTGAGATCGG- 3’	5’-CGGTCAAAGTCTGATGGGTCC- 3’
Slc38a5	5’-CTACAGGCAGGAACGCGAAG- 3’	5’-GGTTGAACACTGACATTCCGA- 3’
β-actin	5’-GGCTGTATTCCCCTCCATCG- 3’	5’-CCAGTTGGTAACAATGCCATGT- 3’

## Results

### Histopathological Changes in the Lungs

**Figure 1:**
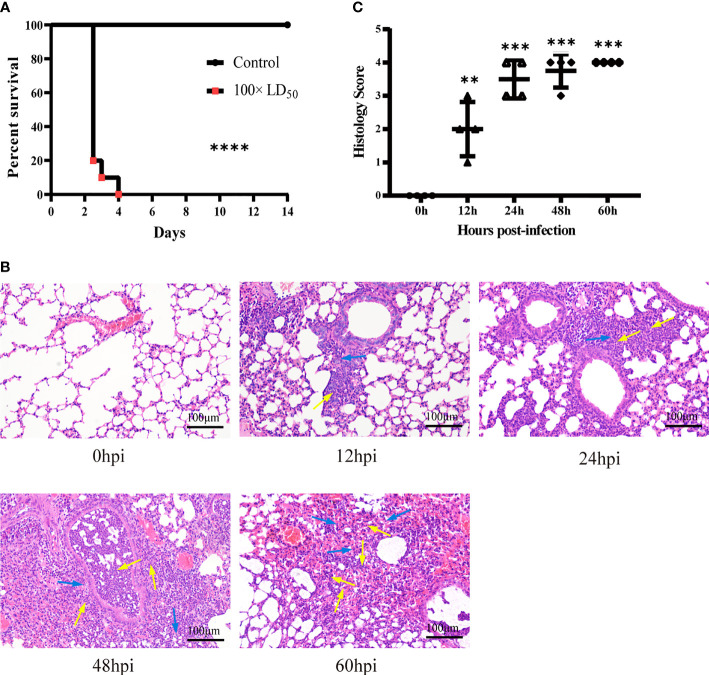
Histopathological analysis of lung tissue from mice infected with hvKp. Mice were challenged with 100 × LD50 (20000 CFU) hypervirulent *Klebsiella pneumoniae* strain (NTUH-K2044), and then the lungs were stained with hematoxylin-eosin (HE). **(A)** Survival curves of mice in the infected and control groups (n = 10 per group). ****p < 0.0001, compared with control group. **(B)** Pathological changes in the lungs at 0, 12, 24, 48 and 60 hours post-infection. Arrows show the infiltration of inflammatory cells; monocytes shown with blue arrows and neutrophils shown with yellow arrows (original magnification = 200×; scale bar = 100 μm). **(C)** Pathological scores of the lung sections; symbols show individual mouse lung scores, horizontal and vertical lines indicate mean and standard deviations of group. Scoring standard: 0, no pathological lesions; 1, minimal; 2, mild; 3, moderate; 4, severe. The degree of pathological lesions was related to the distribution of lesions as follows: inflammatory cell infiltration, edema, congestion, and tissue necrosis. **P < 0.01, ***P < 0.001, compared with 0hpi.

To explore the influence of hvKp on the survival time of mice, we recorded death and survival events for 14 days after infection. Mice started to die in large numbers 2.5 days after infection, with a mortality rate of 80%; at 4 days post-infection, mortality rate was 100% (**Figure 1A**). Therefore, we chose the five time points of 0, 12, 24, 48 and 60 hpi for subsequent experiments and analysis. Histopathological changes in HE-stained lung tissues revealed an intense and continuously increased inflammatory cell infiltration composed of neutrophils and monocytes during the infection (**Figure 1B**, 12-60 hpi). At the last stage of infection (60 hpi), most alveolar cavities were filled with edematous fluid, lung tissue was extensively hemorrhaged and tissue structures were locally destroyed (**Figure 1A**, 60 hpi). These pathological changes were absent in the lungs of control group mice (**Figure 1C**, 0 hpi). Histological scoring also revealed that the severity of lung lesions increased over time (**Figure 1B**). Histopathological validation indicated that hvKp caused excessive acute inflammation and severe lung injury in mice. Hence, the mouse model of acute primary hvKp pneumonia was successfully established, and all subsequent experiments were performed based on this animal model.

### Overview of Transcriptome Analysis

**Figure 2:**
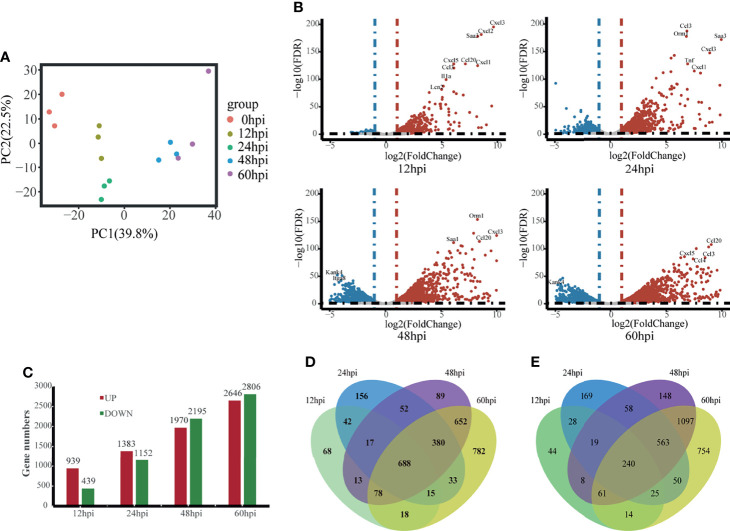
Overview of time-course transcriptome analysis based on the value of FPKM of all groups. **(A)** PCA of the normalized RNA-Seq data of lung tissues in response to hvKp infection. The same color represents the same stages in replicates. **(B)** Volcano plot of RNA-Seq transcriptome data displaying the pattern of gene expression. Significantly differentially expressed genes (DEGs, FDR P ≤ 0.05) are highlighted in red (up-regulated) or blue (down-regulated). Curated genes with specialized biological functions are indicated with labels. **(C)** Number of DEGs at different time points. **(D)** Venn diagram comparing the up-regulated DEGs. **(E)** Venn diagram comparing the Down-regulated DEGs. All transcriptome experiments were performed in biological triplicate.

We isolated hvKp infected lung tissue for RNA-Seq and recorded gene expression profiles at each time point. To assess the quality of the data, principal component analysis (PCA) was used to examine the distribution of samples (**Figure 2A**). The first principal component (PC1) accounted for 39.8% of the total expression variance for the top 1000 most variable genes. The expression matrices of the control and hvKp infected groups separated out along the PC1 axis, with the control group found at one end of the axis, the 12 and 24 hpi groups clustered together distinctly away from the control group, and the 48 and 60 hpi groups also clustered together and even further along the axis away from the control group. Thus, difference in gene expression profiles after hvKp infection indicate an altered transcriptional profile intrinsic to the lung.

Using the control group (0 hpi) as a reference, 6247 DEGs were identified across four time points after hvKp infection. At 12 hpi, 939 upregulated DEGs and 439 downregulated DEGs were detected, and by 60 hpi, 2647 upregulated DEGs and 2806 downregulated DEGs were detected. Overall, the number of upregulated and downregulated DEGs increased over time (**Figures 2B, C**) and a total of 688 genes were upregulated and 240 genes downregulated in common for all time points (**Figures 2D, E**). The fold-change and FDR of the top 20 genes in common with the most significant expression changes in upregulated genes are shown in **Table 2**. Some of these significantly upregulated genes, such as *Csf3*, *Timp1*, *Slc39a14* and *Cxcl3*, are associated with innate immune responses including immune cell differentiation and migration, while some genes, such as *Aoah*, *Slpi* and *Saa1*, are closely associated with inflammatory responses caused by Gram-negative bacteria. Numerous genes significantly upregulated at 12 hpi are associated with inflammatory response, such as chemokines (e.g., *Cxcl3*, *Cxcl2*, *Cxcl5*, *Cxcl1*, *Ccl20* and *Ccl3*), cytokines IL1α and other acute phase molecules (e.g., *Saa3* and *Lcn2*; **Figure 2B**). Genes associated with the control of cytoskeleton formation, such as *Kank4* and *Itga8*, were significantly down-regulated at later stages (≥48 hpi). All results suggest a gradual change in the transcription profile of the lung over time.

**Table 2:** Top 20 DEGs that are significantly up-regulated at different infection time points.

Gene	FDR	12h	24h	48h	60h
		log2FC	log2FC	log2FC	log2FC
Csf3	1.29E-181	9.384	10.128	13.211	14.054
Stfa2	3.57E-139	1.568	3.439	8.466	10.945
F10	8.7E-107	3.022	4.207	5.692	6.375
Timp1	1.78E-106	5.062	5.639	6.890	7.768
Adamts4	8.35E-102	5.304	4.375	7.202	7.675
Saa3	4.66E-101	8.086	10.327	11.928	12.780
Lcn2	3.02E-100	4.949	5.930	7.100	7.274
Igsf6	7.46E-100	1.351	2.215	3.049	3.886
Msr1	2.27E-97	3.005	3.128	4.549	5.081
Slc39a14	1.26E-96	1.572	2.373	3.842	3.939
Slc2a1	1.39E-96	1.043	1.074	3.244	3.995
Aoah	5.65E-95	1.073	2.562	4.070	4.708
AA467197	1.56E-94	3.917	5.505	6.758	7.759
Serpine1	1.35E-93	2.577	2.588	5.616	5.919
Slpi	3.07E-93	2.145	2.242	4.621	4.995
Cxcl3	1.43E-92	9.810	9.150	9.973	10.862
Rtn4rl2	2.92E-90	1.842	2.644	4.270	4.895
Asprv1	4.1E-90	3.039	3.079	4.688	5.791
Saa1	2.5E-88	8.143	8.967	11.773	12.916
Irak3	3.58E-88	1.161	2.381	3.552	4.207

### Analysis of Expression Patterns of DEGs

**Figure 3:**
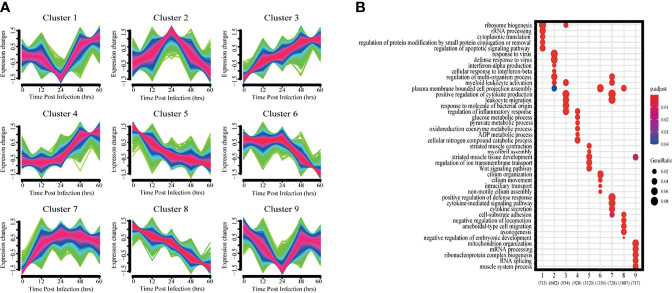
Clustering analysis of expression patterns based on DEGs. **(A)** Clustering by Mfuzz identified nine distinct temporal patterns of gene expression. Red indicates the variation of gene is more conformed to the center of the cluster, followed by blue, and finally green. **(B)** Heatmap shows the significance of the gene ontology (GO) terms in the biological processes describing each of the nine clusters. The number of enriched genes belonging to each cluster is shown in parentheses.

To get a more holistic view, DEGs were clustered into nine clusters according to their temporal expression patterns (**Figure 3A**) and the functional processes associated with each temporal cluster evaluated in a GO enrichment analysis (**Figure 3B**). Few functional processes were commonly enriched, indicating that the gene sets identified by Mfuzz have unique functions. We also found that identified processes were in keeping with the molecular pathophysiology of disease progression.

Clusters 1 and 9 showed a trend of decreasing gene expression levels before reaching a low point at 24 hpi and then increasing. GO analysis showed that 713 and 717 genes in clusters 1 and 9, respectively, were enriched for biological processes related to ribosomes, transcription and translation, including rRNA or mRNA processing, cytoplasmic translation and ribonucleoprotein complex biogenesis.

Cluster 2 gene expression levels peaked at 24 hpi. GO analysis revealed that these 682 genes enrich for biological processes related to interferon immune responses, such as interferon-α production and cellular responses to interferon-β.

Clusters 3 and 7 had gene expression levels that began increasing immediately after infection. GO analysis showed that the 954 and 728 genes in clusters 3 and 7, respectively, were enriched for biological processes related to immune response regulation, including leukocyte activation and migration, positive regulation of cytokine production, cytokine-mediated signaling pathways, and cytokine secretion.

Cluster 4 gene expression levels increased starting at 24 hpi. GO analysis revealed that the 928 genes enrich for biological processes related to glycolysis metabolism, such as glucose, pyruvate, oxidative cofactors and ADP metabolism.

Clusters 5 and 8 gene expression levels decreased after infection. GO analysis revealed that the 1120 and 1887 genes in clusters 5 and 8, respectively, were enriched for biological processes related to normal physiological processes in the organism, including regulation of ion transport across membranes, the Wnt signaling pathway, and cell matrix adhesion.

Cluster 6 gene expression levels decreased continuously after 24 hpi. GO analysis revealed a total of 1350 genes enriched for biological processes associated with ciliary tissue, such as ciliary motility, ciliary transport, and non-motile cilia assembly.

### Functional Modules Identified by Network Analysis

**Figure 4:**
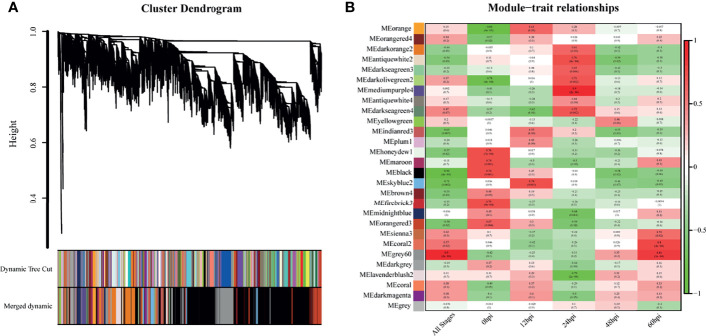
Genes modules in weighted gene co-expression network analysis (WGCNA). **(A)** Hierarchical clustering tree (Cluster Dendrogram) indicates the co-expression modules identified by WGCNA at different infection stages. The branches correspond to modules of highly interconnected groups of genes. The height (y-axis) indicates the co-expression distance and the x-axis corresponds to genes. Colors represent the 28 different modules along with gray indicating genes that could not be assigned to any module. **(B)** A heatmap chart showing module-trait relationships. Red denotes a positive correlation and Blue indicates a negative correlation between the module and infection stage.

WGCNA was performed with the detected DEGs. The dynamic tree cutting algorithm in the WGCNA package was used to process the hierarchical clustering tree, and a total of 28 different modules were finally obtained. The gray modules are the default modules and include discarded genes that could not be clustered, and the rest of the modules were named by randomly assigned colors (**Figure 4A**). Next, we calculated the correlation between the infection process (hpi) and the module genes (**Figure 4B**). To assess the importance of modules for our study, enrichment analysis was performed for modules with correlation coefficients greater than 0.5.

**Figure 5:**
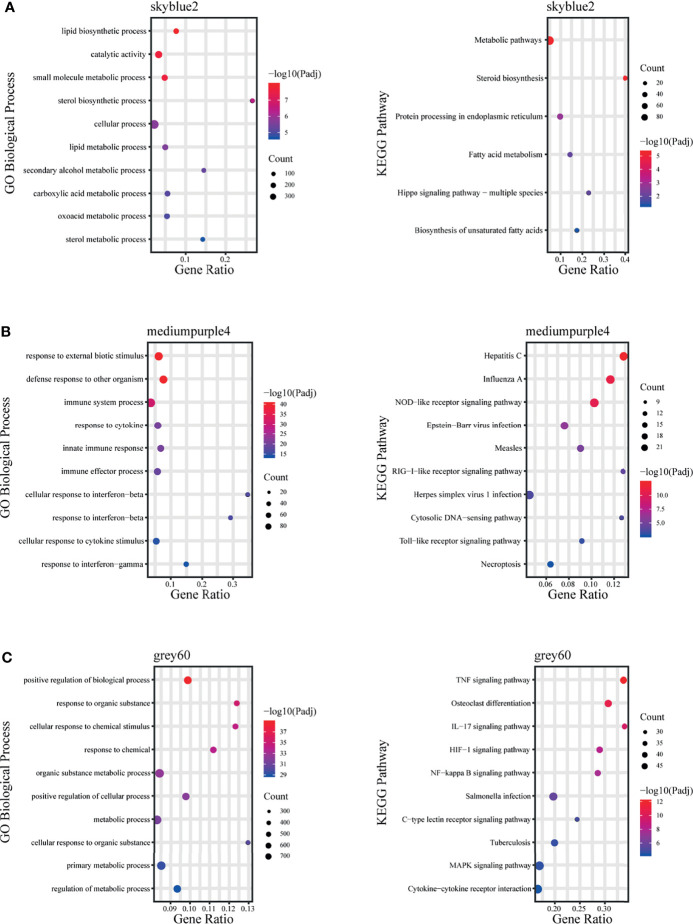
Fold enrichment of top-level overrepresented GO terms (biological process) and KEGG terms within modules that are highly positively correlated with different infection stages. **(A)** skyblue2 module, **(B)** mediumpurple4 module, **(C)** grey60 module.

The skyblue2 module was highly positively correlated with 12 hpi. GO analysis revealed that this module was mainly enriched in lipid-related biological processes including lipid biosynthetic processes, lipid metabolic processes and catalytic activity. KEGG analysis revealed that the main enriched pathways are related to metabolic regulation such as metabolic pathways, steroid biosynthesis and fatty acid metabolism (**Figure 5A**).

The mediumpurple4 module played a key role at 24 hpi. GO analysis revealed that this module was mainly enriched in biological processes related to response to cytokines, including IFN-γ and IFN-β, and in intrinsic immune response to pathogens. KEGG analysis revealed that the main enriched pathways are related to pattern recognition receptors (PRRs), such as NOD-like receptor, RIG-I-like receptor and Toll-like receptor signaling pathways (**Figure 5B**).

The grey60 module was highly positively correlated with all time points, suggesting a key role at all stages. GO analysis revealed that this module was mainly enriched in biological processes, including positive regulation of biological process, response to organic substances and cellular response to chemical stimuli. KEGG analysis revealed enrichment mainly in signaling pathways such as TNF, IL-17, NF-kB, MAPK and HIF-1 (**Figure 5C**).

### Immune Cell Abundance Analysis

**Figure 6:**
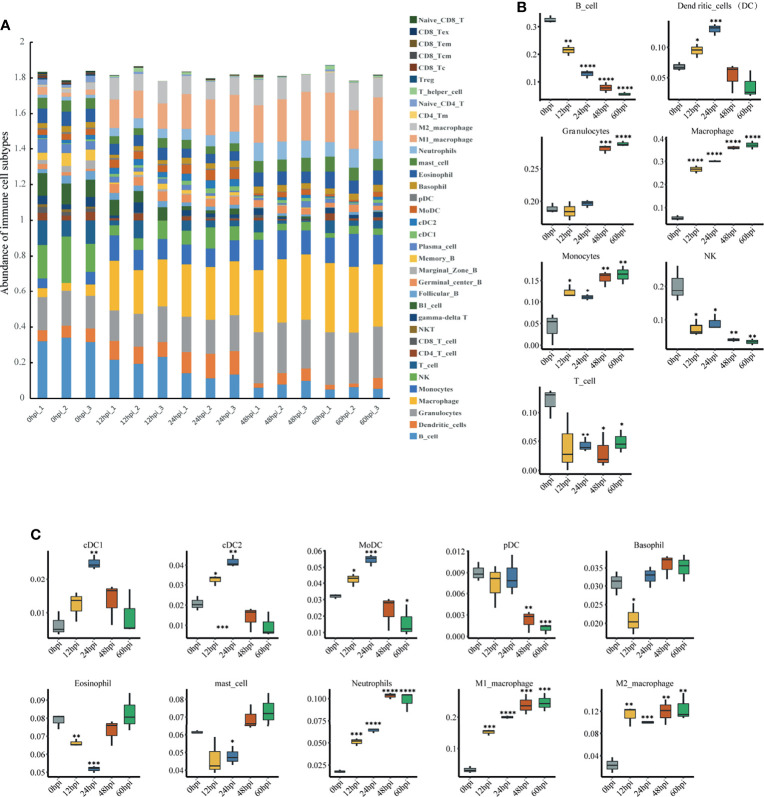
Immune cell infiltration analysis *via* ImmuCellAI-mouse. **(A)** Abundance of 36 immune cell types in all samples. **(B)** Abundance of the seven major immune cell types in layer 1 at different infection stages. **(C)** Abundance of 10 subtypes of the three major innate immune cell types in layer 2 at different infection stages. *P < 0.05, **P < 0.01, ***P < 0.001, ****P < 0.0001, compared with 0 hpi.

To explore immune cell infiltration in lung tissue after infection, we used ImmuCellAI-mouse (Miao et al., 2021) to investigate variation in the infiltration of 36 immune cells among samples from different time points. ImmuCellAI-mouse divided the 36 cell types into three layers using a hierarchical strategy. Abundant immune cell populations with various kinds in each sample were shown in **Figure 6A**. As shown in **Figure 6B**, for the seven major immune cell types in layer 1, an overall decreasing trend with time occurred for B, NK and T cells, while an overall increasing trend occurred for granulocytes, macrophages and monocytes. This suggests innate immunity plays a dominant role after infection. Given this, we further considered the abundance of the 10 subtypes from the three major innate immune cells. Levels of neutrophils, M1 and M2 macrophages increased significantly (**Figure 6C**).

### Quantitative RT-PCR Validation

**Figure 7:**
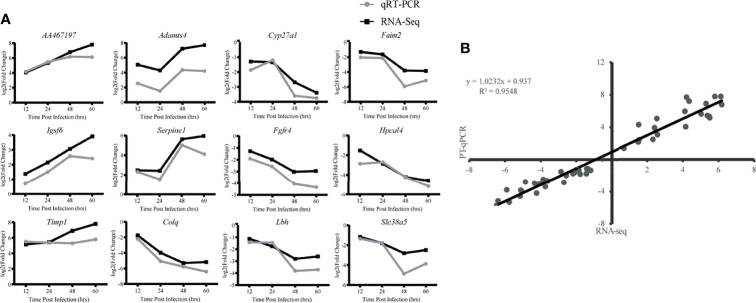
Validation of RNA-Seq data by qRT-PCR. **(A)** Twelve differentially expressed genes **(DEG)** were selected for verification. Relative quantity of gene expression (fold change) for each gene was calculated with the comparative 2^-ΔΔCT^ method. The y-axis shows the fold changes of different infection stages compared to the beginning point, with positive values indicating up-regulation and negative values indicating down-regulation. Each data point was obtained from three biological replicates. **(B)** Correlation of fold change analyzed by data obtained using qRT-PCR (x axis) with RNA-Seq platform (y axis). Correlation analysis was performed using GraphPad software 8.0.

To validate the reproducibility and repeatability of DEGs identified from transcriptome sequencing, 12 DEGs were randomly selected for qRT-PCR validation based on their expression patterns at four time points, namely, *AA467197*, *Adamts4*, *Cyp27a1*, *Faim2*, *Igsf6*, *Serpine1*, *Fgfr4*, *Hpcal4*, *Timp1*, *Colq*, *Lbh* and *Slc38a5* (**Figure 7A**). These genes were significantly differentially expressed and consistently upregulated or downregulated with gene expression changes based on RNA-Seq. Correlation was measured using log2 (fold changes) between RNA-Seq and qRT-PCR; a high correlation coefficient (R^2^) of 0.9548 (**Figure 7B**) confirmed reliability of the transcriptome sequencing data.

## Discussion

Massive inflammation characterized by polymorphonuclear neutrophils and edema is a typical feature of pneumonia in mice caused by *K. pneumoniae* (Bengoechea and Pessoa, 2018). In this study, typical symptoms of pneumonia caused by *K. pneumoniae* were observed in mice within 60 hours after exposure, successfully validating the hvKp pulmonary infection mouse model. Using this model, we then investigated transcriptome profile changes in the lungs of infected mice at different time points to increase our understanding of hvKp pulmonary infection and to screen for genes or targets of research value.

Infection is a dynamic process, and temporal expression pattern clustering can effectively characterize gene expression. To get insight into the gene expression pattern during hvKp pulmonary infection, multiple bioinformatics analysis methods including Mfuzz time clustering, WGCNA and ImmuCellAI-mouse were comprehensively applied in this study. Mfuzz can cluster DEGs from different time points to show their progressive expression dynamics. WGCNA, based on gene co-expression clustering, screens gene modules of interest for further analysis. Recent research has emphasized the potential of this approach for grouping genes into functional modules to reveal the regulatory mechanisms behind complex traits (Behdani and Bakhtiarizadeh, 2017; Mohammad et al., 2018). The ImmuCellAI algorithm can evaluate the abundance of immune cell infiltration in gene expression profiles, and flow cytometry results confirm a higher accuracy compared to other commonly used algorithms, including CIBERSORT and TIMER (Li et al., 2017; Miao et al., 2020; Newman et al., 2015). ImmuCellAI-mouse as a supplement to ImmuCellAI is a comprehensive method for estimating the abundance of immune cells in mouse with a high accuracy (Miao et al., 2021).

At the early stage of infection (12 hpi), an overview of transcriptome analysis showed a rapid increase in the expression of genes encoding pro-inflammatory mediators (chemokines, cytokines and other acute phase molecules), suggesting the triggering of an acute inflammatory response. The skyblue2 module, which was highly associated with 12 hpi in WGCNA analysis, is closely related to the regulation of lipid metabolism, indicating that lipid metabolism plays a key role at 12 hpi. Metabolic regulation is important during host infection and has a significant impact on the immune response (Nhan et al., 2019). Both anti-inflammatory and pro-inflammatory immune responses require energy and metabolic regulation for their development (Kirthana and Ajay, 2014). Thus, to overcome the stresses induced by pathogens, the host needs to regulate relevant metabolic pathways early in the infection. Lipids are not only a very efficient source of energy, but also a key signaling mediator, thus being treated as ‘bioactive lipids’ (Escribá, 2006; Shimizu, 2009). Bioactive lipids play a vital role in immune regulation, inflammation and maintenance of homeostasis within the body (Shimizu, 2009). In the event of tissue infection, innate immune cells are recruited to the infected site and rapidly generate classical eicosanoids, a family of bioactive lipids involved in immunity and inflammation, whose main roles include amplifying or reducing inflammation, coordinating leukocyte recruitment, cytokine and chemokine production, antibody formation, cell proliferation and migration, and antigen presentation (Funk, 2001; Nathan, 2002; Harizi et al., 2008). Accordingly, we speculate that lipid metabolism may reflect a pro-inflammatory effect in the early stage of hvKp pulmonary infection and may have some clinical significance. However, further studies are needed to fully understand the underlying mechanisms.

Cluster2, whose gene expression level peaked at 24 hpi in the Mfuzz analysis, and the mediumpurple4 module, which is highly correlated with 24 hpi, are both closely associated with interferons according to GO analysis. The KEGG analysis for the mediumpurple4 module associates it with PRRs signaling pathways. Cellular immunity is essential to clear pathogens and type II interferon IFN-γ is a key molecule in promoting cellular immunity. A complex interaction between immune cell activity and IFN-γ leads to initiation of a cascade of pro-inflammatory responses through coordinated integration of other signals involving cytokines and PRRs, such as TNF-α, LPS and type I interferons (Kak et al., 2018). IFN-γ is an essential activator of antibacterial macrophages (Nathan, 1983). Glycolytic metabolism, which also promotes the survival, differentiation, and effector function of activated macrophages (Van den Bossche et al., 2015), had a sustained rise in the expression of its associated genes in cluster 4 starting from 24 hpi. Correspondingly, macrophage abundance in ImmuCellAI-mouse analysis continued to increase at 24 hpi after an already significant increase.

In the immune cell infiltration analysis, neutrophils and monocytes consistently increased after infection. Although neutrophil migration and activation are essential for infection clearance, excessive neutrophil recruitment and aberrant activation can lead to severe host tissue injury and may ultimately lead to death (Weber et al., 2015). This was reflected in our pathological findings, where after lung immune cells were significantly activated and recruited, local damage to lung tissue structures followed at 48 hpi. However, the abundance of NK cells continued to decrease. This finding is contrary to a previous study that found no change in NK cells in the lung during the first two days after *K. pneumoniae* infection (Xu et al., 2014). It is also dissimilar to responses observed with most bacteria, such as *Streptococcus pneumoniae* (van den Boogaard et al., 2016), *Shigella flexneri* (Le-Barillec et al., 2005), *Pseudomonas aeruginosa* (Wesselkamper et al., 2008), and *Staphylococcus aureus* (Small et al., 2008), where NK cells remain constant or increase after pulmonary infection. This suggests the decrease in pulmonary NK cells may be specific to hvKp infection. NK cells account for the highest percentage of resident lymphocytes in the lung, unlike other tissues (Wang et al., 2012; Sun et al., 2013), suggesting that NK cells are essential for the pulmonary immune response when pathogens invade. Although NK cells are traditionally well known for their critical protective role in antiviral innate immunity (Lodoen and Lanier, 2006), growing evidence indicates that NK cells are closely associated with fighting bacterial infections, with both beneficial or detrimental effects on the organism possible. For example, Dunn and North (Dunn and North, 1991) showed that early production of IFN-γ by NK cells is essential for resistance to *L. monocytogenes*. NK cells also contribute to host defense against *K. pneumoniae* (Xu et al., 2014). In contrast, other studies have shown that NK cell depletion leads to enhanced clearance of *L. monocytogenes* (Teixeira and Kaufmann, 1994) and a significant increase in survival for *Streptococcus pneumoniae* lung-infected mice (Christaki et al., 2015). These contrasting results could be related to the diversity of infection routes, bacterial strains and mouse strains and this possibility should be followed up. Interestingly, NK cells are also found to be reduced in paraquat dichloride induced lung injury, and *in vivo* NK cell depletion reduces macrophage and neutrophil infiltration, resulting in attenuated lung injury (Wu et al., 2020). Therefore, we hypothesize that hvKp lung infection decreases NK cells for attenuated inflammatory cell infiltration. The role of NK cells in severe hvKp lung infections, which could provide new targets for therapy, deserves further investigation and clarification.

During infection biological processes associated with immune response regulation, such as leukocyte activation and migration, cytokine production, cytokine-mediated signaling pathways and cytokine secretion were continuously activated. While biological processes associated with normal physiological processes of the organism, such as the regulation of ion transport across the membrane, the Wnt signaling pathway and cell matrix adhesion were continuously inhibited. This suggests that the organism undergoes a violent and sustained inflammatory response after infection and that the structures of the lungs are damaged. The grey60 module was highly positively correlated with all infection time points, indicating that the module genes play a key role in the infection process. KEGG analysis showed that grey60 module genes were closely associated with TNF, IL-17, HIF-1, NF-kB, and MAPK signaling pathways. TNF and IL-17 signaling pathways have important functions in host defense and disease pathogenesis (Gaffen, 2009; Kalliolias and Ivashkiv, 2016). NF-kB and MAPK are signaling pathways help mediate lung inflammatory responses (Lavoie et al., 2010; Wang et al., 2013). LPS is a potent stimulator that triggers MAPK and NF-kB signaling pathways (Li et al., 2015; Zhang et al., 2015). NF-kB is a key target of the p38 MAPK signaling pathway after LPS binding of TLR4/MYD88 to initiate the intracellular pathway (Shi et al., 2019; Zhou et al., 2019). P38MAPK/MK2 can regulate LPS-induced gene expression by controlling NF-kB p65 hyperphosphorylation and nuclear translocation (Gorska et al., 2007; Ehlting et al., 2011). Given the prominent role of these signaling pathways in hvKp pulmonary infection, further elucidation of the mechanisms of these signaling pathways in hvKp immunopathogenesis could provide novel insights for emergency treatment.

Some of the top 20 upregulated genes during the infection process are associated with the innate immune response, while others are closely associated with the inflammatory response induced by Gram-negative bacteria. One of them, *Saa1* encodes serum amyloid A1 (SAA1), an acute phase protein in the inflammatory response and can be an antimicrobial agent by acting as a direct modulator of bacteria (Buck et al., 2016; Sun and Ye, 2016). Inducible expression of SAA1 in the acute phase may protect the host from Gram-negative bacterial infection by reducing LPS-induced tissue damage (Cheng et al., 2017). *Lcn2* encodes Lipocalin-2 (LCN2), a key antimicrobial protein, whose primary antibacterial function is to bind and sequester bacterial iron carriers, thereby depriving bacteria of the iron that provides them with nutrients (Goetz et al., 2002). Previous work found LCN2 protein is upregulated in the lung after *K. pneumoniae* infection (Chan et al., 2009), and our findings support this. *Aoah* encodes a mammalian enzyme named acyloxyacyl hydrolase (AOAH) that can inactivate LPS in host tissues (Munford et al., 2009) and promotes recovery from lung inflammation caused by *K. pneumoniae* (Zou et al., 2017). *Slpi* encodes secretory leucocyte peptidase inhibitor (SLPI) that is secreted from lung tissue, exhibits antibacterial and antifungal properties and is an essential respiratory host defense protein similar to antimicrobial peptides (Gomez et al., 2009; Majchrzak-Gorecka et al., 2016). *Irak3* encodes interleukin-1 receptor-associated kinase 3 (IRAK3; also known as IRAK-M), which has been rarely studied in bacterial pulmonary infections. However, IRAK3 is known to be involved in the regulation of LPS tolerance (van ‘t Veer et al., 2007; Yu et al., 2017), whereby cells or organisms exposed to LPS enter a non-responsive state and are unable to respond to further LPS stimuli, thus preventing inflammatory overload (Biswas and Lopez-Collazo, 2009; Hotchkiss et al., 2013). IRAK-M mRNA was upregulated in the lungs of WT mice with *K. pneumoniae* (Hoogerwerf et al., 2012) and our findings are consistent with this. Among these five genes involved in the antimicrobial response to bacterial infection, *Saa1*, associated with the LPS cell wall component of Gram-negative bacteria that causes host inflammatory response, and *Slpi*, which encodes an antimicrobial protein, have not previously been reported in hvKp lung infection. Given their persistently high expression levels and multiple functions in hvKp infected lungs, they are expected to be good therapeutic targets for intervention in hvKp-induced lung injury and deserve to be investigated in depth.

Our findings suggest that hvKp causes a primary acute inflammation in the lung that increases over time, leading to the damage of lung structures. Clarifying the role of NK cells in severe hvKp lung infections could provide new targets for therapy. *Saa1* and *Slpi*, significantly upregulated during infection and associated with the immune response, have not been reported in hvKp infections and could be important targets for subsequent studies. Given that disease progression may depend on immune cells, further explorations at the level of a single cell type are warranted to elucidate the cellular associations of the phenomena found in this study. In conclusion, our work both validates the results of previous studies on *K. pneumoniae* pulmonary infections and provides new insights on hvKp pulmonary infections that have promise for the development of therapeutic approaches to reduce hvKp lung inflammation.

## Data Availability Statement

The data presented in the study are deposited in the Gene Expression Omnibus (GEO) repository, accession number GSE199546. The original contributions presented in the study are publicly available. This data can be found here: https://www.ncbi.nlm.nih.gov/geo/query/acc.cgi?acc=GSE199546.

## Ethics Statement

The animal study was reviewed and approved by Animal Care and Use Committee, Academy of Military Medical Science.

## Author Contributions

DZ, TF, and WY conceived and designed the experiments. XZ, CC, TQ, LH, and LZ performed the experiments. XZ, JG, YZ, XS, and ML analyzed and interpreted the results. XZ and JG wrote the manuscript. All authors contributed to the article and approved the submitted version.

## Conflict of Interest

The authors declare that the research was conducted in the absence of any commercial or financial relationships that could be construed as a potential conflict of interest.

## Publisher’s Note

All claims expressed in this article are solely those of the authors and do not necessarily represent those of their affiliated organizations, or those of the publisher, the editors and the reviewers. Any product that may be evaluated in this article, or claim that may be made by its manufacturer, is not guaranteed or endorsed by the publisher.
